# The Role and Function of Non-Coding RNAs in Cholangiocarcinoma Invasiveness

**DOI:** 10.3390/biomedicines13061369

**Published:** 2025-06-03

**Authors:** Yu Meng, Fang Wei, Ye Zhang, Wenting He, Haijiao Yan, Jun Wu

**Affiliations:** Department of Oncology, The Third Affiliated Hospital of Soochow University, Changzhou 213000, China; 19856263346@163.com (Y.M.);

**Keywords:** non-coding RNAs, miRNAs, lncRNAs, circRNAs, invasiveness, EMT, migration and invasion

## Abstract

Cholangiocarcinoma (CCA) is an aggressive tumor that originates from the epithelial cells of the bile duct and has the ability to metastasize to the liver or lymph nodes at an early stage. CCA metastasis represents a complex, multi-stage cascade process. Among these stages, the acquisition of invasiveness by CCA cells is a critical prerequisite for metastatic progression. Elucidating the molecular mechanisms driving CCA cell invasiveness is critical for advancing our knowledge in this field. Emerging evidence highlights the critical role of non-coding RNAs (ncRNAs), including microRNAs (miRNAs), long non-coding RNAs (lncRNAs), and circular RNAs (circRNAs). These molecules orchestrate key processes such as the epithelial–mesenchymal transition (EMT), as well as the migration and invasion of CCA cells. Collectively, these processes ultimately drive tumor progression. This review comprehensively synthesizes the expression, biogenesis, interactions, signaling pathways, and functional mechanisms of ncRNAs in the invasiveness of CCA. Furthermore, the review discusses potential clinical applications of ncRNAs, including their roles as diagnostic tools, therapeutic targets, and prognostic markers. These investigations offer novel insights and evidence for identifying early metastasis in CCA, developing specific therapeutic strategies, and enhancing drug resistance.

## 1. Introduction

CCA is a malignant tumor originating in the biliary tract, accounting for approximately 3% of all gastrointestinal malignancies [1]. Based on anatomical location, CCA can be categorized into three distinct subtypes: intrahepatic (iCCA), perihilar (pCCA), and distal (dCCA) cholangiocarcinoma. ICCA arises proximal to the second-order bile ducts; in contrast, extrahepatic cholangiocarcinoma (eCCA) is subdivided into pCCA and dCCA, with the cystic duct serving as the anatomical boundary [1,2]. The advent of second- and third-generation sequencing technologies has facilitated the identification of numerous genetic alterations in CCA. ICCA frequently harbors alterations such as IDH1/2 mutations and FGFR2 gene fusions, whereas KRAS and BRAF mutations are more prevalent in eCCA. Moreover, high-frequency mutations in tumor suppressor genes—including *TP53*, *ARID1A*, and *SMAD4*—as well as in genes associated with chromatin remodeling and epigenetic dysregulation, are widely found in both iCCA and eCCA [3,4]. Over the past four decades, the global incidence and mortality rates associated with CCA have demonstrated a consistent upward trajectory, with significantly elevated rates observed in East Asia and Southeast Asia compared to other regions. For instance, the incidence rate in Thailand can reach as high as 85 per 100,000 person-years, whereas in Western countries, it ranges from merely 0.5 to 3.4 per 100,000 person-years [5]. The etiology of CCA varies across different regions: in Western nations, primary sclerosing cholangitis (PSC) is recognized as the predominant carcinogenic factor, whereas in Asian countries, parasitic infections and intrahepatic bile duct stones are more commonly encountered [2]. The diagnosis of CCA frequently occurs at an advanced stage, primarily due to the limited availability of specific and sensitive biomarkers, the anatomical inaccessibility of the bile ducts, and the nonspecific nature of its clinical symptoms. These factors collectively contribute to delayed detection, often resulting in missed opportunities for early surgical intervention. For patients diagnosed with early-stage CCA, surgical resection remains the sole treatment modality that may provide a potential cure. Nevertheless, even following radical surgical intervention, the prognosis for CCA remains unfavorable. Evidence suggests that the recurrence rate within the first year following surgery can reach an alarming 67%. In stark contrast, the five-year survival rate remains dismally low at approximately 2% [6]. The dismal survival rate among patients diagnosed with CCA is profoundly associated with its substantial metastatic potential, as intrahepatic and lymph node metastases frequently develop during the early stages of the disease [7].

**Figure 1:**
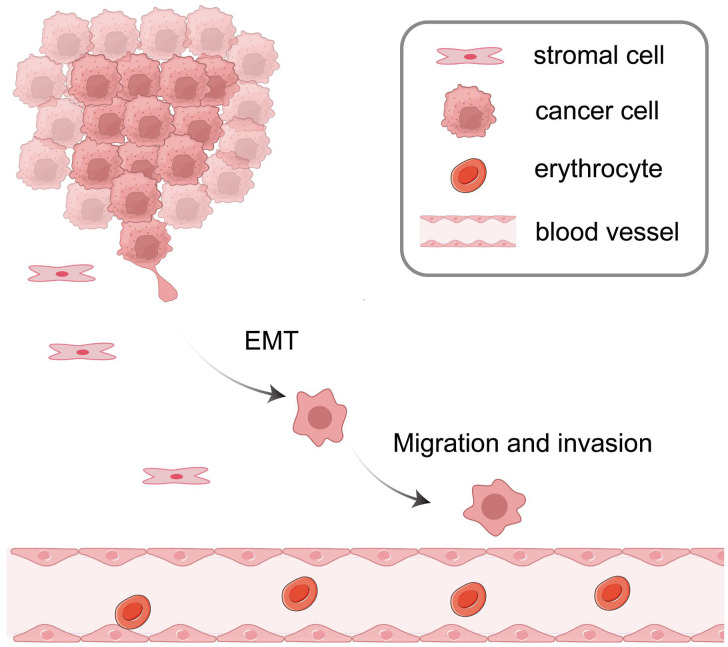
EMT, migration, and invasion in CCA progression. Initially, cancer cells undergo EMT, which facilitates a phenotypic alteration that enhances their migratory capabilities. Subsequently, these cancer cells migrate and invade adjacent tissues, which may ultimately result in metastatic dissemination.

The acquisition of invasiveness by tumor cells signifies the initial phase of the metastatic cascade, a process intricately associated with EMT. EMT represents a pivotal biological process in which epithelial cells, including both normal and cancerous subtypes, transition from an epithelial phenotype to a mesenchymal phenotype, acquiring enhanced migratory and invasive properties. This cellular transition endows enhanced motility, increased invasiveness, and the ability to degrade the extracellular matrix (ECM). These characteristics are essential for the invasion and metastatic dissemination of tumor cells [8,9]. EMT represents a dynamic and multifaceted process characterized by coordinated alterations at the molecular, structural, and functional levels. These alterations encompass the disruption of cell polarity and intercellular adhesion, downregulation of epithelial markers such as E-cadherin, and upregulation of mesenchymal markers including N-cadherin and Vimentin. Furthermore, cytoskeletal remodeling plays a critical role in facilitating the morphological transformations associated with EMT [10,11]. Accumulating evidence indicates that EMT plays a critical role in the malignant progression of CCA. Pan et al. demonstrated that extracellular vesicles derived from Clonorchis sinensis contribute to the malignant proliferation and metastasis of CCA by inducing EMT via activation of the NF-κB and ERK signaling pathways [12]. Furthermore, Oba et al. analyzed immunohistochemical (IHC) staining of C-C chemokine receptor 7 (CCR7) in 181 patients with perihilar cholangiocarcinoma and found that CCR7 expression was significantly correlated with the mesenchymal cell phenotype and facilitated migration and invasion of extrahepatic cholangiocarcinoma cells by inducing EMT [13]. However, the mere occurrence of EMT is insufficient to fully realize the entire invasion–metastasis process. The migratory and invasive behaviors exhibited by CCA cells are also contingent upon the regulation of various signaling pathways and genes (Figure 1). These signaling pathways and genes are crucial for the regulation of cellular migratory and invasive processes, thereby enabling CCA cells to flourish in complex microenvironments and successfully disseminate to distant organs.

The human genome harbors a significant quantity of ncRNAs, which constitute approximately 90% of its total genomic composition [14]. Within the human genome, only 1–2% of the DNA is responsible for encoding proteins. Historically, 98% of non-coding sequences were regarded as “transcriptional noise” that does not encode proteins. Nevertheless, accumulating evidence indicates that, despite ncRNAs being highly conserved in comparison to protein-coding genes, the majority of ncRNAs are functional. These molecules constitute intricate and cryptic molecular genetic signals, which serve crucial roles in modulating and orchestrating an array of physiological activities as well as pathological mechanisms [15]. ncRNAs are commonly classified according to their length into three categories: small ncRNAs (<50 nucleotides (nt)), medium ncRNAs (50–200 nt), and long ncRNAs (>200 nt). Small ncRNAs mainly comprise miRNAs, piRNAs, and siRNAs. Medium ncRNAs predominantly include rRNAs, tRNAs, snRNAs, and snoRNAs. In contrast, long ncRNAs mainly consist of lncRNAs and circRNAs, since the majority of circRNAs are longer than 200 nt [16] (Table 1). In recent years, advancements in whole-genome sequencing and high-throughput transcriptome analyses have unveiled the regulatory potential of ncRNAs, particularly in the context of tumor progression and metastasis. This review primarily concentrates on the extensively studied miRNAs, lncRNAs, and circRNAs, examining their influence on the invasiveness of CCA cells through the regulation of key processes such as EMT, migration, and invasion. This study conducts an in-depth analysis of the molecular mechanisms underlying the functions of ncRNAs, with the goal of improving the understanding of the invasive characteristics of CCA cells, identifying biomarkers indicative of early metastasis, and establishing molecular targets for targeted therapies designed to prevent the distant dissemination of CCA cells.

## 2. Overview of ncRNAs

### 2.1. Overview of miRNAs

**Figure 2:**
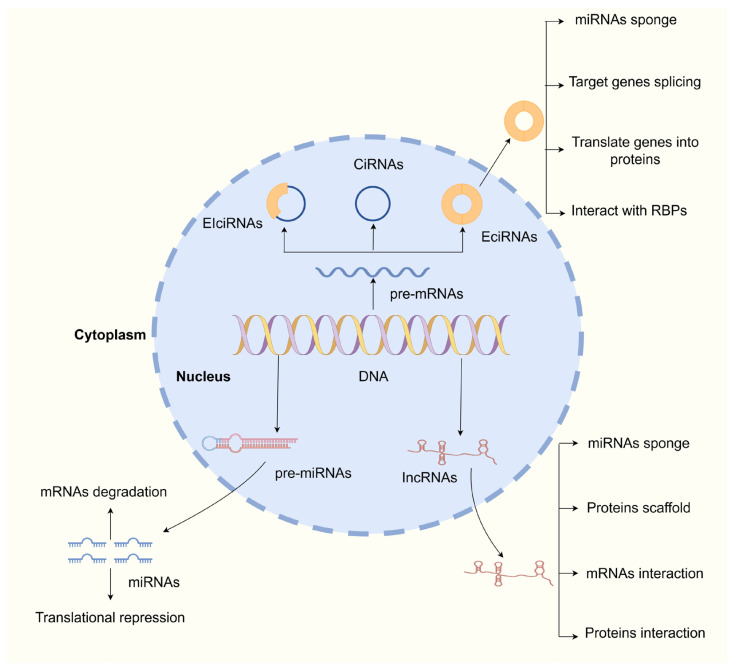
Biogenesis and function of ncRNAs. DNA is transcribed within the cell nucleus to produce various precursor ncRNAs, including pre-miRNAs and pre-mRNAs. These precursor RNAs undergo further processing within both the nucleus and cytoplasm, resulting in the production of diverse functional ncRNAs, including miRNAs, lncRNAs, and circRNAs. These mature ncRNAs subsequently execute a variety of cellular regulatory functions.

miRNAs represent a class of short, non-coding RNA molecules, typically ranging from 17 to 25 nt in length, and are found in diverse biological contexts, including cells, exosomes, and bodily fluids such as blood and saliva [17]. miRNAs exert a multitude of biological effects in tumorigenesis and progression, encompassing chemotherapy resistance, inflammation, migration and invasion, proliferation, apoptosis, differentiation, and angiogenesis [18]. Primary miRNAs (pri-miRNAs) are transcribed from miRNA genes and subsequently processed through a multi-step mechanism that includes nuclear splicing, export to the cytoplasm, and Dicer-mediated cleavage, ultimately resulting in the generation of mature double-stranded miRNAs. Activated miRNAs interact with Argonaute (AGO) proteins to form the miRNA-induced silencing complex (miRISC). The miRISC complex specifically recognizes and binds to the 3′ untranslated region (UTR) of target mRNAs, thereby inducing either mRNA degradation or translational repression, ultimately leading to gene silencing (Figure 2) [19]. miRNAs recognize mRNAs through imperfect base pairing, wherein the 5′ end of miRNAs demonstrates a high degree of sequence complementarity with the 3′ UTR of target mRNAs. This binding region is relatively short, necessitating only 6 to 8 base pairs, while permitting a certain degree of imperfect pairing [20]. This distinctive characteristic enables a single miRNA to simultaneously interact with and regulate multiple mRNAs, thereby playing a pivotal role in a variety of biological functions, including proliferation, migration, invasion, and apoptosis [21,22].

### 2.2. Overview of lncRNAs

lncRNAs represent a class of RNA molecules exceeding 200 nt in length and are significantly enriched in the cytoplasm relative to the nucleus when compared to protein-coding genes [23]. lncRNAs typically contain at least one open reading frame (ORF) for potential protein translation; however, the ORFs of the majority of lncRNAs remain untranslated. Even when a limited number are translated into proteins, their protein products are highly unstable and subject to rapid degradation [24]. Consequently, lncRNAs were historically regarded as non-functional transcriptional byproducts or transcriptional noise. Recent evidence indicates that lncRNAs represent pivotal molecular regulators capable of profoundly modulating tumorigenesis and cancer progression through non-canonical mechanisms independent of protein-coding potential. For example, lncRNAs function as a miRNA sponge, protein scaffold, mRNAs interaction, and protein interaction [25]. lncRNAs have been shown to modulate gene expression across three distinct levels: pre-transcriptional, transcriptional, and post-transcriptional. At the pre-transcriptional level, lncRNAs regulate gene expression through histone modifications, gene alterations, and chromatin remodeling. During transcription, lncRNAs regulate gene expression by influencing the activity of transcription factors. At the post-transcriptional stage, lncRNAs influence gene expression through histone modifications, gene alterations, and chromatin remodeling [26]. Their competitive binding as competing endogenous RNAs (ceRNAs) to miRNAs constitutes a crucial mechanism through which lncRNAs participate in various physiological processes and tumorigenesis (Figure 2) [27].

### 2.3. Overview of circRNAs

circRNAs have recently gained attention as an exciting and emerging area of research within the field of ncRNAs. circRNAs can be systematically classified into three distinct categories based on their origin and structural characteristics: exonic circRNAs (EciRNAs), intronic circRNAs (CiRNAs), and exon-intron circRNAs (EIciRNAs) [28]. Among these, CiRNAs and EIciRNAs are primarily enriched in the nucleus and directly participate in transcriptional regulation, whereas EciRNAs are predominantly located in the cytoplasm, where they function as ceRNAs to sponge miRNAs and regulate translation at the post-transcriptional level [29] (Figure 2). circRNAs are produced through processes such as exon skipping or back-splicing, leading to the creation of covalently closed circular structures [30]. Due to the absence of 3′ and 5′ ends, this distinctive structure exhibits enhanced stability, rendering it less susceptible to degradation by ribonucleases (RNAse) and resulting in a half-life that exceeds 48 h. In contrast, mRNAs exhibit a relatively short half-life of approximately 10 h, resulting in circRNAs being over tenfold more abundant than their linear counterparts within the cytoplasmic compartment, thereby indicating their significant potential in cancer detection [31]. Numerous studies have shown that circRNAs play a significant role in regulating gene transcription and translation to promote tumorigenesis, with mechanisms including acting as a miRNA sponge, targeting genes splicing, translating genes into proteins, and interacting with RNA-binding proteins (RBPs) [32,33]. Recent investigations have challenged the longstanding assumption that circRNAs exclusively serve as non-coding elements, revealing that numerous circRNAs harbor internal ribosome entry sites (IRESs) and ORFs. These features confer the capacity for translation into functional peptides or proteins [34,35]. Notably, a study has shown that IL-6 stimulates the upregulation of circGGNBP2, which encodes a peptide designated cGGNBP2-184aa. This peptide promotes phosphorylation at tyrosine 705 (Tyr705) of the signal transducer and activator of STAT3, thereby augmenting STAT3 signaling pathways and facilitating the proliferation and metastasis of ICC cells both in vitro and in vivo [36].

## 3. ncRNAs in EMT

### 3.1. miRNAs in EMT

EMT is a critical biological process that facilitates cancer cell invasion and metastasis. This transformation involves the downregulation of epithelial markers, such as E-cadherin, alongside the upregulation of mesenchymal markers, including N-cadherin and Vimentin. The process is tightly regulated by a complex network of EMT-associated transcription factors (EMT-TFs), such as Snail, Slug, ZEB1/2, and Twist1. Recent studies underscore the critical role of miRNAs in orchestrating EMT regulation and modulating the associated transcription factors that govern this process.

MiR-200b is a key regulator in inhibiting EMT and reducing the invasive potential of CCA cells. Zhu et al. demonstrated that this is achieved through the modulation of EMT markers and suppression of the STAT3 signaling pathway [37]. Similarly, miR-34a inhibits the invasive potential of CCA cells by attenuating EMT induced by the TGF-β/Smad4 signaling pathway [38]. The miR-30 family, particularly miR-30e, plays a significant role in regulating EMT transcription factors by directly targeting the 3′ UTR of Snail. This interaction suppresses Snail expression and inhibits TGF-β-induced EMT, ultimately reducing the migratory and invasive abilities of CCA cells [39]. Likewise, miR-186 suppresses Twist1 expression and inhibits EMT through interaction with Twist1 mRNA’s 3′ UTR [40]. MiR-204 suppresses EMT by directly targeting Slug, facilitating a shift from mesenchymal to epithelial states and markedly decreasing cell migration and invasion [41]. In a rat model of iCCA, exosomes transport miR-195 to iCCA cells, specifically aiming at and suppressing Snail expression. This action effectively hinders the process of EMT, highlighting its crucial influence within the tumor microenvironment [42].

By contrast, some miRNAs accelerate the progression of CCA by enhancing EMT. MiR-221 significantly downregulates E-cadherin expression while simultaneously boosting N-cadherin and MMP2 levels via the β-catenin/c-Jun signaling pathway. This mechanism amplifies the invasive and migratory potential of CCA cells, driving their aggressive behavior [43]. MiR-329 facilitates EMT in CCA cells by decreasing E-cadherin levels while increasing the expression of N-cadherin and Vimentin; furthermore, the interaction between miR-329 and LAMB3 elucidates its pivotal role in modulating EMT processes [44]. MiR-19b-3p targets CCDC6, disrupting its inhibitory effect on β-catenin nuclear translocation. This activation of the β-catenin signaling pathway subsequently increases Snail expression, thereby accelerating EMT progression in iCCA cells [45]. Elevated levels of miR-21 facilitate EMT by suppressing E-cadherin expression and inducing the expression of N-cadherin and Vimentin, as well as upregulating Snail and Slug through the AKT/ERK1/2 signaling pathway, ultimately enhancing the invasive capacity of CCA cells [46].

### 3.2. lncRNAs in EMT

In the invasion and metastasis of CCA, EMT serves as a key biological process characterized by disrupted cell–cell adhesion and enhanced invasive capacity [47,48]. lncRNAs act as important regulatory factors, precisely controlling the EMT process and EMT-TFs through diverse mechanisms. On one hand, LINC01503 [49], LINC00667 [50], LINC00261 [51], H19 [52], and PCAT1 [53] directly downregulate the epithelial marker E-cadherin while upregulating mesenchymal markers N-cadherin and Vimentin, promoting EMT progression in CCA cells. On the other hand, CCAT1 relieves ROCK2 inhibition by competitively binding to miR-181a-5p [54], while LINC00665 acts as a molecular sponge for miR-424-5p to upregulate BCL9L and activate the Wnt/β-catenin signaling pathway, both promoting EMT through ceRNA mechanisms [55]. Furthermore, CCAT2 demonstrates cross-cancer type EMT regulatory capabilities, regulating Snail2 expression in hepatocellular carcinoma [56], interacting with epigenetic factors EZH2, H3K27me3, and LSD1 in gastric cancer [57], and modulating EMT-related gene expression in CCA [58]. LncRNA ATB exerts an opposing mechanism by acting as a ceRNA and interacting with miR-200c to weaken its inhibitory effect on ZEB1 and ZEB2 [59]. This leads to an increased expression of ZEB1 and ZEB2, thereby initiating and accelerating the EMT process. Additionally, lncRNA ZEB1-AS1 can serve as a molecular sponge for miR-200a, reducing the miR-200a-mediated suppression of ZEB1. Consequently, ZEB1 expression is markedly elevated, promoting the transition of cells to a mesenchymal phenotype [60]. Together, these form a complex regulatory network of lncRNAs controlling EMT progression in CCA, providing potential molecular targets for diagnosis and treatment of CCA.

However, some lncRNAs exert inhibitory effects on EMT and thus suppress cell invasion and metastasis. For instance, lncRNA MEG3 significantly inhibits the EMT process in CCA cells by directly targeting and suppressing the expression of the Snail gene [61].

### 3.3. circRNAs in EMT

Increasing evidence demonstrates that circRNAs serve as pivotal regulators of EMT, highlighting their essential role in this process. For instance, circ_0058106 acts as a ceRNA for miR-153 to modulate Snail1 expression, thereby promoting the nuclear translocation of Twist1 and initiating the EMT process, ultimately enhancing the invasive capacity of laryngeal cancer cells [62]. Meanwhile, circRNA_0023642 in gastric cancer cells promotes EMT by upregulating N-cadherin, Vimentin, and Snail while simultaneously suppressing E-cadherin [63]. In colorectal cancer, circSKA3 facilitates EMT by preventing the ubiquitination and degradation of Slug [64]. Although substantial progress has been made in understanding how circRNAs regulate EMT, their role in CCA remains insufficiently explored. Evidence suggests that circ_0059961 plays a crucial role in modulating the migratory and invasive behaviors of CCA cells by influencing EMT-associated genes. This highlights the broader significance of circRNAs in EMT regulation and tumor invasiveness within CCA [65].

## 4. ncRNAs in CCA Cell Migration and Invasion

### 4.1. miRNAs

During the progression of CCA, miRNAs play a pivotal role in enhancing the migratory and invasive potential of CCA cells. They drive malignant progression by modulating downstream genes and critical signaling cascades, including PI3K/AKT, Wnt/β-catenin, Notch, and JAK/STAT pathways. By orchestrating these mechanisms, they serve as critical drivers of tumor initiation, growth, and progression [66,67]. Chen et al. reported that miR-129-2-3p is significantly downregulated in both tumor tissues and corresponding cell lines of iCCA. Its reduced expression is strongly associated with unfavorable pathological features, such as distant metastasis, poor differentiation, and advanced TNM stages. Further studies demonstrated that miR-129-2-3p directly targets the 3′ UTR of Wip1, leading to decreased Wip1 activity and subsequently inhibiting the migration and invasion of CCA cells [68]. Wip1 is an oncogene localized to the chromosomal region 17q22-q23 that facilitates the migration and invasion of iCCA cells by upregulating MMPs expression and suppressing p53 activity [69]. Furthermore, miR-1182 and miR-let-7a act synergistically to downregulate NUAK1, thereby limiting the migration, invasion, and proliferation of CCA cells while concurrently promoting autophagy [70]. NUAK1 regulates cellular energy homeostasis and facilitates the proliferation and survival of tumor cells [71].

Beyond regulating downstream genes that govern CCA cell migration and invasion, miRNAs intricately modulate pivotal signaling pathways. For instance, miR-144 primarily acts as a tumor suppressor by inhibiting the AKT signaling pathway and directly targeting LIS1, which leads to the suppression of CCA cell proliferation and invasion [72]. Similarly, miR-885-5p suppresses the PI3K/AKT signaling pathway by directly targeting GALNT3, which, in turn, reduces metastasis in iCCA [73]. MicroRNAs predominantly target the PI3K/AKT signaling cascade as a critical regulatory mechanism in CCA cell migration and invasion dynamics. These molecular interactions effectively modulate cellular motility and invasive potential. Our findings illuminate the fundamental role of the PI3K/AKT signaling pathway in governing CCA cellular behavior, providing crucial mechanistic insights into cancer progression and potential therapeutic strategies.

In the Wnt/β-catenin signaling pathway, Wnt activation triggers the stabilization and accumulation of β-catenin in the cytoplasm, allowing it to translocate into the nucleus. Once in the nucleus, β-catenin functions as a co-activator for transcription factors, thereby regulating the expression of target genes involved in various cellular processes [74]. MiR-let-7c modulates the aggressive behavior of CCA cells by directly targeting DVL3, leading to the suppression of the DVL3/β-catenin signaling pathway [75]. An increasing body of research has demonstrated that multiple miRNAs, including miR-26b-5p [76], miR-7-5p [77], miR-144-5p and miR-451a [78], miR-320 [79], and miR-373 [80], also exhibit tumor-suppressive effects in CCA, inhibiting the migration and invasion of CCA cells.

In contrast to tumor-suppressive miRNAs, specific miRNAs demonstrate pronounced oncogenic roles in driving CCA progression. Farnesoid X Receptor (FXR) plays a crucial role in regulating cholesterol, bile acid, and glucose metabolism. As a nuclear receptor, FXR modulates the expression of genes involved in these metabolic pathways, maintaining homeostasis and protecting against metabolic disorders [81]. Interestingly, abnormal bile acid metabolism is closely associated with CCA. MiR-421, an oncogenic miRNA, inhibits FXR expression by binding to the FXR 3′UTR, thereby promoting the migration and invasion of CCA cells [82]. Extracellular vesicles (EVs) are membrane-bound entities secreted by cells that enable the intercellular transfer of miRNAs, thereby contributing to the malignant progression of CCA [83,84]. MiR-210, an oncogenic miRNA, triggers G2/M phase arrest in CCA cells at the G2/M phase and confers resistance to gemcitabine [85]. EVs derived from CCA transfer miR-210 to CCA cells, where it directly targets the RECK 3′UTR, thereby suppressing RECK expression and facilitating the growth, metastasis, and chemoresistance of CCA [86]. Recent studies have demonstrated that multiple miRNAs, including miR-21 [87], miR-96 [88], miR-383 [89], miR-24 [90], and miR-122-5p [91], also promote cell migration and invasion in CCA cells, indicating that these miRNAs may have a substantial regulatory role in tumor metastasis.

### 4.2. lncRNAs

lncRNAs regulate miRNA expression through the ceRNA network. By functioning as molecular sponges, lncRNAs sequester miRNAs, effectively modulating their availability and profoundly impacting the migration, invasion, and aggressiveness of CCA cells [92]. TTN-AS1 acts as a molecular sponge for miR-513a-5p, effectively suppressing its expression. This interaction upregulates the target gene SFN, a critical driver of CCA cell proliferation and migration, ultimately promoting tumor progression [93]. SFN is a protein that plays a critical role in cell cycle regulation and cellular stress responses, and has been implicated in the initiation and progression of multiple malignant tumors [94,95]. Further supporting these findings, INC00976 acts as a ceRNA through its interaction with miR-3202, which upregulates GPX4 expression, inhibits ferroptosis, and consequently enhances the proliferation, migration, and invasion of CCA cells [96]. Moreover, research has shown that lncRNA PKD2-2-3 competitively interacts with miR-328, reducing its suppression of GPAM. This interaction results in increased GPAM expression, thereby boosting the proliferation and invasion of CCA cells while concurrently inhibiting apoptosis [97]. GPAM encodes a mitochondrial enzyme that catalyzes the rate-limiting step in triglyceride biosynthesis and plays a crucial role in the regulation of lipid metabolism [98].

Notably, FOXD2-AS1 promotes the migration and invasion of CCA cells by functioning as a molecular sponge for miR-760, leading to the upregulation of E2F3 expression. Additionally, high FOXD2-AS1 expression is significantly associated with advanced TNM stages, lymph node metastasis, and poorer survival outcomes in CCA patients [99]. Likewise, LMCD1-AS1 enhances the proliferation and invasion of CCA cells via the miR-345-5p/COL6A3 axis, concurrently decreasing cellular apoptosis [100]. LINC00184 also exhibits significant oncogenic effects. Functioning as a ceRNA for miR-23b-3p, LINC00184 upregulates ANXA2 expression, consequently promoting the proliferation, invasion, and migration of CCA cells while significantly impacting adenine metabolism [101].

Previous studies have highlighted the indispensable role of lncRNAs in orchestrating diverse signaling pathways. For instance, NEAT1 boosts the expression of PTP4A1 by functioning as a molecular sponge for miR-186-5p, thereby stimulating the PI3K/AKT signaling pathway, which plays a crucial role in the proliferation, migration, and invasion of CCA cells [102]. Moreover, in CCA, the Hippo pathway governs cell proliferation, migration, invasion, and apoptosis via its effector protein YAP1. Research has demonstrated that PAICC activates the Hippo pathway, which is crucial for the progression of CCA [103]. STAT3 acts as a critical junction for various oncogenic signaling pathways, significantly influencing the regulation of immune responses in tumors. Research has shown that the JAK/STAT pathway becomes overly active as CCA develops and advances [104]. Furthermore, LOXL1-AS1 has been demonstrated to enhance the proliferation, migration, and invasion of CCA cells by activating the JAK2/STAT3 signaling pathway [105].

In contrast to the pro-migration effects described above, another study has revealed that MT1JP suppresses the migration and invasion of iCCA cells by inactivating the Wnt/β-catenin signaling pathway through the miR-18a-5p/FBP1 axis [106].

### 4.3. circRNAs

In CCA, circRNAs are crucial regulators of tumor migration and invasion through various molecular mechanisms, such as the ceRNA network and signaling pathways, exhibiting dual regulatory functions. Certain circRNAs exhibit tumor-suppressive properties by inhibiting migration and invasion. For example, circNFIB directly interacts with the N-terminal domain (NTD) of MEK1, thereby suppressing the activation of the MEK1/ERK2 signaling pathway and reducing ERK2 phosphorylation levels. This mechanism effectively limits the spread and infiltration of iCCA cells both in vitro and in vivo. Additionally, increased circNFIB expression in iCCA has been shown to delay the development of resistance to trametinib [107].

Nevertheless, increasing evidence indicates that circRNAs predominantly exhibit oncogenic properties in CCA. Research has demonstrated that circSLCO1B3 promotes the proliferation, migration, and invasion of CCA cells by activating the SMAD3 and TGF-β signaling pathways through the miR-502-5p/HOXC8 axis [108]. Moreover, circZNF215 is markedly upregulated in metastatic iCCA tissues following surgery, and its expression level correlates with unfavorable patient prognosis. Mechanistic investigations indicate that circZNF215 disrupts the interaction between PRDX1 and PTEN, resulting in the oxidative inactivation of PTEN, which subsequently activates the PI3K/AKT signaling pathway to facilitate tumorigenesis [109]. In addition, circRAPGEF5 functions as a molecular sponge for miR-3185, stabilizing SAE1 expression, which promotes the SUMOylation of AKT, thereby enhancing the proliferation, migration, and invasion of iCCA cells [110]. Importantly, circCDR1as binds to miR-641, accelerating its degradation and diminishing the inhibitory effect of miR-641 on AKT3 and mTOR, thus promoting the proliferation and invasion of CCA cells [111]. Furthermore, circACTN4 plays a critical role in the progression of iCCA by activating the Hippo pathway through the miR-424-5p/YAP1 axis and recruiting YYBX1 to enhance FZD7 transcription. This dual mechanism synergistically activates the Wnt/β-catenin pathway, driving tumor progression [112].

## 5. Perspectives and Conclusions

CCA is recognized as a highly aggressive malignancy characterized by a poor prognosis, restricted therapeutic options, and an absence of reliable biomarkers for early diagnosis. Over the past few decades, despite the gradual increase in the global incidence and mortality rates of CCA, significant challenges remain in elucidating its molecular mechanisms and developing effective therapeutic strategies [113,114]. Over the past decade, a growing body of research has unveiled the pivotal role of ncRNAs as master regulators of diverse physiological and pathological processes. Recent advances in high-throughput technologies and bioinformatics have enabled the comprehensive profiling of ncRNAs expression, leading to the discovery of critical ncRNAs linked to tumor aggressiveness.

**Figure 3:**
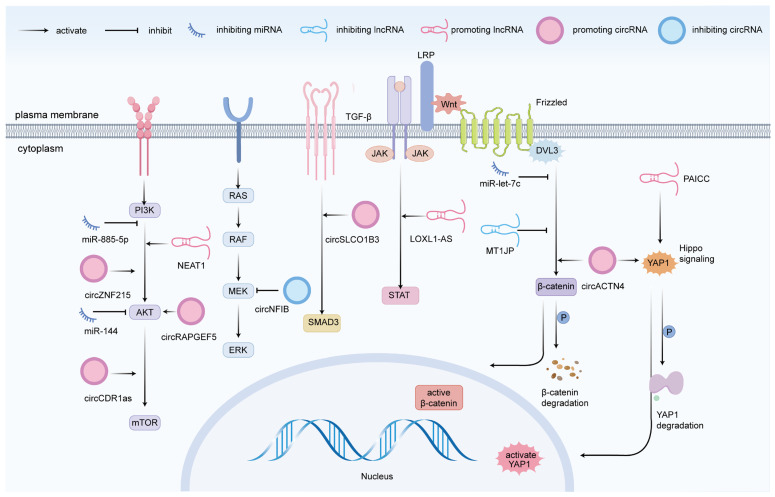
Schematic diagram of ncRNA-involved pathways in CCA cell migration and invasion. These pathways include PI3K/AKT, RAS/RAF/MEK/ERK, TGF-β, JAK/STAT, Wnt/β-catenin, and Hippo.

EMT is a pivotal process that enables CCA cells to acquire invasiveness. During EMT, tumor cells undergo a transition from a stationary epithelial state to a motile and invasive mesenchymal phenotype. This transformation is accompanied by matrix-degrading capabilities. Cells in the mesenchymal state form invasive pseudopods, breach the basement membrane, and detach from the primary tumor. These changes ultimately facilitate their distant dissemination and metastasis [115,116]. However, EMT alone is insufficient for CCA cells to complete the entire invasion–metastasis cascade; it also relies on multiple signaling pathways or genes involved in migration and invasion. Current research on ncRNAs predominantly emphasizes miRNAs, lncRNAs, and circRNAs, among which miRNAs are particularly active throughout the invasion–metastasis cascade. Through a systematic and comprehensive literature review, we first summarize the biogenesis of miRNAs, lncRNAs, and circRNAs, as well as their functions and mechanisms in tumorigenesis and progression. Subsequently, we focus on the regulatory mechanisms of these three types of ncRNAs in key steps of CCA cell invasiveness, such as EMT, migration, and invasion (Table 2). We further investigate the intricate signaling pathways modulating the migratory and invasive phenotypes of CCA cells, governed by miRNAs, lncRNAs, and circRNAs (Figure 3). This approach establishes a robust foundation for comprehensively elucidating the molecular mechanisms underlying CCA invasion and metastasis.

In addition to EMT, the sustained self-renewal of CCA cells is a critical prerequisite for the acquisition of invasive phenotypes. In recent years, the hypothesis that only cancer cells with stem-like self-renewal capacity are capable of driving metastatic dissemination has garnered significant attention in oncological research. For instance, Hermann et al. discovered that CD133^+^ CXCR4^+^ cancer stem cells (CSCs) in pancreatic tumors are predominantly located at the invasive front, where they play a crucial role in determining the invasive phenotype of cancer cells [117]. Similarly, Pang et al. demonstrated in colorectal cancer that the presence of CD26^+^ CSCs predicts distant metastasis and enhanced invasiveness [118]. These studies highlight the critical role of CSCs in acquiring invasive phenotypes and initiating tumor metastasis. Subsequent analyses demonstrate that the let-7c/miR-99a/miR-125b cluster significantly suppresses stem cell-like characteristics by downregulating CD133 and CD44 via inhibition of the IL-6/STAT3 signaling pathway, thereby markedly reducing the invasiveness and tumor-initiating potential of CCA cells [119]. Conversely, lncRNA PKD2-2-3 is highly expressed in CCA CSCs, and its overexpression not only upregulates CSC markers CD44 and CD133 but also enhances the spheroid-forming ability and chemotherapy resistance of CCA cells [120]. This indirectly suggests that ncRNAs participate in regulating the overall invasiveness of CCA cells by modulating the properties of CSCs.

Advances in bioinformatics, genomics, and proteomics are rapidly elucidating the complex roles of ncRNAs in CCA progression and pathogenesis. These ncRNAs demonstrate considerable promise as diagnostic biomarkers, novel therapeutic targets, and potential agents for addressing chemoresistance mechanisms in clinical applications. Recent studies have demonstrated that miR-150 and miR-200c may serve as potential diagnostic biomarkers, distinguishing CCA from other hepatic disorders with high sensitivity and specificity. Furthermore, exosomal miR-21 and miR-1246 have been identified as promising biomarkers for the early detection of CCA [66]. MiR-21 and miR-221 demonstrate diagnostic utility for hepatolithiasis-associated CCA, achieving high accuracy (AUC = 0.911), with sensitivity and specificity values of 77.42% and 97.50%, respectively [121]. Serum miR-150-5p expression is downregulated in CCA patients, exhibiting a diagnostic sensitivity of 91.43% and specificity of 80%. When combined with CA19-9 expression, sensitivity increased to 93.33% and specificity to 96.88% [122]. Xu et al. reported that CCA patients with elevated circ-CCAC1 levels in both bile- and serum-derived EVs displayed robust diagnostic performance. The diagnostic value of serum EVs (AUC = 0.759) was comparable to that of serum CA19-9 (AUC = 0.757), whereas bile EVs (AUC = 0.857) exhibited superior performance to CA19-9. Notably, combining EV-circ-CCAC1 (from bile or serum) with serum CA19-9 enhanced diagnostic performance compared to either marker alone. Furthermore, the study confirmed that elevated circ-CCAC1 expression (*p* = 0.001) serves as an independent prognostic marker for iCCA and predicts postoperative recurrence in iCCA patients (*p* = 0.002) [123]. Zhou et al. demonstrated that elevated lncRNA TTN-AS1 levels are associated with lymph node metastasis in CCA and indicative of reduced survival rates [124]. Gemcitabine, a first-line chemotherapeutic agent for CCA, frequently elicits drug resistance in patients. miR-206 suppresses gemcitabine resistance in CCA cells by disrupting interactions between cancer cells and cancer-associated fibroblasts (CAFs) [125]. Although accumulating preclinical evidence highlights the potential of ncRNAs as therapeutic targets for CCA, their clinical translation remains constrained by tumor heterogeneity, the complexity of the in vivo microenvironment, and multifaceted ncRNA functionalities, necessitating further investigation.

RNA sequencing (RNA-seq), developed from advances in DNA sequencing technologies, has revolutionized the analysis and discovery of ncRNAs through next-generation sequencing (NGS) and third-generation long-read sequencing platforms, largely superseding traditional microarray methods. Due to its high sensitivity and broad dynamic range, NGS enables comprehensive detection of both known and novel ncRNAs, with distinct advantages in identifying low-abundance species. Notably, third-generation sequencing technologies such as PacBio SMRT and Oxford Nanopore provide ultra-long-read sequences, effectively overcoming the limitations of traditional short-read sequencing in transcript assembly and offering critical technical support for the identification of novel ncRNAs with complex secondary structures. Numerous studies have demonstrated that RNA-seq is a powerful tool for identifying and analyzing dysregulated ncRNAs in complex diseases, including cancer and cholangiocarcinoma, thereby establishing a foundation for elucidating their biological functions and potential as biomarkers [126,127]. However, due to the limited availability of clinical samples from CCA patients, the mechanisms of related ncRNAs in CCA have not been thoroughly investigated. Whether interactions exist among ncRNAs and whether crosstalk occurs between signaling pathways remain unclear. The role of ncRNAs in tumor cells’ invasiveness is intricate and multifaceted, with ncRNAs functioning as either oncogenic or tumor-suppressive. Interestingly, some ncRNAs demonstrate dual roles, varying across different studies, tumor types, or even within the same tumor context. For example, lncRNA MALAT1 demonstrates opposing roles in different studies and tumor types. In esophageal squamous cell carcinoma, MALAT1 acts as an oncogene [128]. In contrast, in laryngeal squamous cell carcinoma, MALAT1 inhibits tumor cell proliferation, migration and invasion [129]. These contradictions may arise from differences in research methodologies, the diverse effects on signaling pathways in different cancer contexts, and complex interactions with other genes. This diversity and contradiction pose challenges for ncRNAs as therapeutic targets. Therefore, it is crucial to conduct more in-depth research on the specific mechanisms of ncRNAs at various stages of CCA invasion to fully understand their functions in this process. Understanding the regulatory mechanisms of ncRNAs in specific contexts provides an important theoretical foundation for developing effective therapeutic targets.

## Figures and Tables

**Table 1:** Length-based classification of ncRNAs.

Category	Length	Representative Types
Short ncRNAs	<50 nt	miRNAs, siRNAs, piRNAs
Medium ncRNAs	50–200 nt	rRNAs, tRNAs, snRNAs, snoRNAs
Long ncRNAs	>200 nt	lncRNAs, circRNAs

**Table 2:** ncRNAs associated with CCA invasiveness.

ncRNA	Assessed Cell Line	Targets or Modulated Molecules	Pro/Anti	References
miRNA				
miR-200b	HIBEpiC, TFK-1, HuCCT-1	STAT3 pathway	Anti-EMT	[37]
miR-34a	QBC939, HuCCT1	TGF-β/Smad4 pathway	Anti-EMT	[38]
miR-30e	HuCCT1, HuH28, OZ	Snail, TGF-β pathway	Anti-EMT	[39]
miR-186	CCLP1, SG-231, HIBEC	Twist1	Anti-EMT	[40]
miR-204	ICC-9810, RBE, HuH28, HuCCT1	Slug	Anti-EMT	[41]
miR-195	human: LX2, HuCCT1, SG231, TFK1, H69; rat:BDEne, BDEsp, RGF	Snail	Anti-EMT	[42]
miR-221	QBC 939, HuCCT1	PTEN, β-catenin/c-Jun pathway	Pro-EMT	[43]
miR-329	RBE	LAMB3	Pro-EMT	[44]
miR-19b-3p	HUCCT1, RBE, CCLP-1, TFK-1, HIBEpiC	CCDC6, Snail, β-catenin pathway	Pro-EMT	[45]
miR-21	QBC939	AKT/ERK1/2 pathway, Snail, Slug	Pro-EMT	[46]
lncRNA				
LINC01503	RBE and QBC939	-	Pro-EMT	[49]
LINC00667	CCLP-1, QBC939, RBE, HCCC-9810, HIBEC	miR-200c-3p/PDK1	Pro-EMT	[50]
LINC00261	QBC939, RBE	-	Pro-EMT	[51]
H19	QBC939, RBE	-	Pro-EMT	[52]
PCAT1	RBE, HCCC-9810, CCLP-1, QBC939, HIBEC	miR-216a-3p/BCL3	Pro-EMT	[53]
LINC00665	HuCCT1, HuH28, SNU-1196, SNU-1079, SNU-308, SNU-245, SNU-478, SNU-869, HEK293T	miR-424-5p/BCL9L, Wnt/β-Catenin pathway	Pro-EMT	[54]
CCAT1	HCC-9810, RBE	miR-181a-5p/ROCK2	Pro-EMT	[55]
CCAT2	RBE, HCCC-9810, QBC939, CCLP-1, Huh-28, HuCCT1, HIBEC	-	Pro-EMT	[58]
ATB	BEC, HUCCT1, RBE, TFK1, Huh-28	miR-200c, ZEB1, ZEB2	Pro-EMT	[59]
ZEB1-AS1	HuH28, HuCCT1, RBE, CCLP-1, HCCC-9810, HIBEC	miR-200a, ZEB1	Pro-EMT	[60]
MEG3	QBC939, TFK-1, HCCC-9810, RBE, CCLP-1, HIBEC	Snail’	Anti-EMT	[61]
circRNA				
circ_0059961	CCLP-1, QBC939, HIBEC	miR-629-5p/SFRP2	Anti-EMT	[65]
miRNA				
miR-129-2-3p	QBC-939, RBE, BEC	Wip1	Anti-migration and invasion	[68]
miR-1182andmiR-let-7a	HIBEPIC, CCC-5, HCC-9810, Huh28	NUAK1	Anti-migration and invasion	[70]
miR-144	HCCC-9810, CCLP1, HuCC-T1, RBE	LIS1, AKT pathway	Anti-migration and invasion	[72]
miR-885-5p	HuCCT1, RBE, Huh28	GALNT3, PI3K/AKT pathway	Anti-migration and invasion	[73]
miR-let-7c	TFK-1, HUCCT-1	EZH2, DVL3/β-catenin pathway	Anti-migration and invasion	[75]
miR-26b-5p	RBE, HCCC-9810	S100A7	Anti-migration and invasion	[76]
miR-7-5p	HCCC-9810, HuCCT1, QBC-939, RBE, HIBEC	MyD88	Anti-migration and invasion	[77]
miR-144-5p and miR-451a	HuCCT-1, HCCC 9810, RBE, TFK-1	ST8SIA4	Anti-migration and invasion	[78]
miR-320	CCLP-1, QBC939, HIBEC	NRP-1	Anti-migration and invasion	[79]
miR-373	QBC939, HIBEpic	MBD2	Anti-migration and invasion	[80]
miR-421	HCCC-9180, SSP25, RBE, GBC-SD, HEK293T	FXR	Pro-migration and invasion	[82]
miR-210	KKU-213, KKU-055, KKU-100	RECK	Pro-migration and invasion	[86]
miR-21	M213, M214, KKU100, M055, M139, M156, OCA17, MMNK1	PDCD4	Pro-migration and invasion	[87]
miR-96	HuCCT1, HuH28, RBE, HIBEC	MTSS1	Pro-migration and invasion	[88]
miR-383	RBE, HuCCT1, QBC939, CCLP, HIBEpic	IRF1	Pro-migration and invasion	[89]
miR-24	Mz-ChA-1, TFK-1, SG231, CCLP-1, HuCC-T1, HuH-28	MEN1	Pro-migration and invasion	[90]
miR-122-5p	HIBEpiC, QBC939, LIPF155C, LICCF, CCLP1, RBE, HEK293T	ALDOA	Pro-migration and invasion	[91]
lncRNA				
TTN-AS1	HIBEC, TFK-1, CCLP, HCCC-9810, HUCCT1	miR-513a-5p/SFN	Pro-migration and invasion	[93]
LINC00976	HIBEC, HuCCT1, HCCC-9810, QBC939, HuH28, RBE	miR-3202/GPX4	Pro-migration and invasion	[96]
PKD2-2-3	HuH28, HuCCT1, RBE, TFK1, HIBEpiC	miR-328/GPAM	Pro-migration and invasion	[97]
FOXD2-AS1	HIBEC, CCLP-1, QBC939, HuCCT1, RBE	miR-760/E2F3	Pro-migration and invasion	[99]
LMCD1-AS1	RBE, KMBC, QBC939, HCCC-9810, HuCCT1, HIBEC	miR-345-5p/COL6A3	Pro-migration and invasion	[100]
LINC00184	KMBC, HuCCT1, QBC939, HIBEC	miR-23b-3p/ANXA2	Pro-migration and invasion	[101]
NEAT1	HuCCT1, RBE, HCCC-9810, HCCCT-1	miR186-5p/PTP4A1, PI3K/AKT pathway	Pro-migration and invasion	[102]
PAICC	QBC-939, HUCCT-1, HCCC-9810, HIBEC	miR-141-3p and miR-27a-3p/YAP1, Hippo pathway	Pro-migration and invasion	[103]
LOXL1-AS1	HIBEC, CCLP-1, QBC939, RBE, HuCCT1	miR-324-3p/ABCA1, JAK2/STAT3 pathway	Pro-migration and invasion	[105]
MT1JP	HCCC-9810, RBE, HUCCT1	miR-18a-5p/FBP1, Wnt/β-catenin pathway	Anti-migration and invasion	[106]
circRNA				
circNFIB	HuCCT1, HCCC9810, RBE	MEK1/ERK2 pathway	Anti-migration and invasion	[107]
circSLCO1B3	RBE, CCLP1, HCCC, HIBEpic	miR-502-5p/HOXC8, TGF-β/SMAD3 pathway	Pro-migration and invasion	[108]
circZNF215	HuCCT1, RBE, HCCC9810	PRDX1, PTEN, PI3K/AKT pathway	Pro-migration and invasion	[109]
circRAPGEF5	RBE, CCLP1, 9810, HUCCT1, HIBEC	miR-3185/SAE1, AKT pathway	Pro-migration and invasion	[110]
circCDR1as	HIBEpiC, HCCC-9810, RBE, HIBEpiC	miR-641/AKT3/mTOR	Pro-migration and invasion	[111]
circACTN4	HIBEpiC, RBE, QBC939, FRH0201	miR-424-5p/YAP1, Hippo pathway, Wnt/β-catenin pathway	Pro-migration and invasion	[112]

## Data Availability

Data are contained within the article.

## Abbreviations

The following abbreviations are used in this manuscript:

piRNAs	Piwi-interacting RNAs
siRNAs	Small interfering RNAs
rRNAs	Ribosomal RNAs
tRNAs	Transfer RNAs
snRNAs	Small nuclear RNAs
snoRNA	Small nucleolar RNAs
Snail	Snail family transcriptional repressor 1
Slug	Snail family transcriptional repressor 2
Twist1	Twist-related protein 1
ZEB1/2	Zinc finger E-box-binding homeobox 1/2
LAMB3	Laminin subunit beta 3
CCDC6	Coiled-coil domain containing 6
BCL9L	B-cell CLL/lymphoma 9-like
EZH2	Enhancer of zeste homolog 2
H3K27me3	Histone H3 lysine 27 trimethylation
LSD1	Lysine-specific demethylase 1
Wip1	Wild-type p53-induced phosphatase 1
NUAK1	NUAK family kinase 1
GALNT3	N-acetylgalactosaminyl transferase-3
S100A7	S100 calcium-binding protein A7
MyD88	Myeloid differentiation primary response 8
ST8SIA4	ST8 alpha-N-acetyl-neuraminide alpha-2,8-sialyltransferase 4
NRP-1	Neuropilin-1
RECK	Reversion-inducing cysteine-rich protein with Kazal motifs
PDCD4	Programmed cell death protein 4
MTSS1	Metastasis suppressor 1
IRF1	Interferon regulatory factor 1
MEN1	Multiple endocrine neoplasia type 1
ALDOA	Aldolase A
SFN	Stratifin
GPX4	Glutathione peroxidase 4
GPAM	Glycerol-3-phosphate acyltransferase
E2F3	E2F transcription factor 3
COL6A3	Collagen type VI alpha 3 chain
ANXA2	Annexin A2
PTP4A1	Protein tyrosine phosphatase 4A1
FBP1	Fructose-1,6-bisphosphatase 1
YAP1	Yes-associated protein 1
HOXC8	Homeobox C8
PRDX1	Peroxiredoxin 1
PTEN	Phosphatase and tensin homolog
SAE1	SUMO-activating enzyme subunit 1
SUMO	Small ubiquitin-like modifier
YBX1	Y-Box binding protein 1
FZD7	Frizzled-7
